# Cost-effectiveness of a reactive oral cholera immunization campaign using Shanchol™ in Malawi

**DOI:** 10.1186/s12962-021-00270-y

**Published:** 2021-03-10

**Authors:** Patrick G. Ilboudo, Martin A. Mengel, Bradford D. Gessner, Bagrey Ngwira, Philippe Cavailler, Jean-Bernard Le Gargasson

**Affiliations:** 1 Agence de Médecine Préventive, 10 BP 638, Ouagadougou, Burkina Faso; 2 Agence de Médecine Préventive, 21 boulevard Pasteur, Paris, 75015 France; 3 Pfizer Inc, Collegeville, PA USA; 4 The Polytechnic, Chichiri 3, Blantyre, Malawi; 5 Agence de Médecine Préventive, Bureau Ferney-Voltaire, Bat. JB Say, 4e, aile A, 13, chemin du Levant, Ferney-Voltaire, 01210 France

**Keywords:** Cost-effectiveness, Cholera, Shanchol, Model, Malawi

## Abstract

**Background:**

Oral cholera vaccines (OCV) have been recommended as additional measures for the prevention of cholera. However, little is known about the cost-effectiveness of OCV use in sub-Saharan Africa, particularly in reactive outbreak contexts. This study aimed to investigate the cost-effectiveness of the use of OCV Shanchol in response to a cholera outbreak in the Lake Chilwa area, Malawi.

**Methods:**

The Excel-based Vaccine Introduction Cost-Effectiveness model was used to assess the cost-effectiveness ratios with and without indirect protection. Model input parameters were obtained from cost evaluations and epidemiological studies conducted in Malawi and published literature. One-way sensitivity and threshold analyses of cost-effectiveness ratios were performed.

**Results:**

Compared with the reference scenario i.e. treatment of cholera cases, the immunization campaign would have prevented 636 and 1 020 cases of cholera without and with indirect protection, respectively. The cost-effectiveness ratios were US$19 212 per death, US$500 per case, and US$738 per DALY averted without indirect protection. They were US$10 165 per death, US$264 per case, and US$391 per DALY averted with indirect protection. The net cost per DALY averted was sensitive to four input parameters, including case fatality rate, duration of immunity (vaccine’s protective duration), discount rate and cholera incidence.

**Conclusion:**

Relative to the Malawi gross domestic product per capita, the reactive OCV campaign represented a cost-effective intervention, particularly when considering indirect vaccine effects. Results will need to be assessed in other settings, e.g., during campaigns implemented directly by the Ministry of Health rather than by international partners.

**Supplementary Information:**

The online version contains supplementary material available at 10.1186/s12962-021-00270-y.

## Introduction

Cholera remains endemic in many areas of the world that have poor water, hygiene and sanitation access, and unsafe food practices, particularly sub-Saharan Africa [1–3]. Over the long-term, improvements in water, hygiene, and sanitation will reduce cholera burden [4, 5]. Yet, these will take decades to achieve in the world’s poorest communities [6]. Oral cholera vaccines (OCV) have been prequalified by WHO and may be used as an interim and synergistic measure to mitigate cholera disease burden [7].

There is an abundance of literature demonstrating the protective efficacy of the low-cost Shanchol vaccine [8–12]. Because of this, recent years have witnessed an increased routine utilization of Shanchol oral cholera vaccine in settings where cholera is endemic. More notable was the increased usage of the aforementioned vaccine to rapidly mitigate ongoing cholera outbreaks in emergency contexts such in Lake Chilwa, owing to its reduced cost per dose [13].

From December 2015 to August 2016, 1 256 cholera cases were notified in the area surrounding Lake Chilwa, mainly in fishing communities. In response, the Malawian Ministry of Health, supported by the World Health Organization (WHO) and international partners, including Agence de Médecine Préventive and Médecins sans Frontières, conducted a reactive vaccination campaign in three administrative districts, including Machinga, Phalombe, and Zomba in addition to strengthening surveillance, case management and water and sanitation improvements. All three districts had an estimated total population of 1,895,625 inhabitants in 2016. The vaccination campaign targeted in total 90,000 individuals aged more than one-year-old, including pregnant women. The first round of the campaign was held between 16 and 22 February 2016 and begun the same day in the three settings. The second round of the campaign was conducted between 8 and 15 March 2016.

The costs of the reactive vaccination campaign and vaccine delivery per dose, as well as household and health facility costs for treating cholera have already been published by the authors [14, 15]. However, little is known about the cost-effectiveness of Shanchol vaccine use in sub-Saharan Africa, including during reactive outbreak contexts. The aim of the present study was to investigate the cost-effectiveness of Shanchol vaccine use in response to the outbreak in Malawi.

## Methods

### Ethics statement

The cost-effectiveness analysis was part of the campaign monitoring and evaluation activities approved by the MoH and partners, including the Polytechnic in Blantyre, Médecins Sans Frontières and the French Agence de Médecine Préventive. Participants provided written informed consent prior to interviews.

### Population and setting

The OCV campaign was implemented in Machinga, Phalombe, and Zomba. During 2014, the three Malawian districts had an estimated combined population of 1,733,250 inhabitants [16]. Health services were mainly delivered to these communities through health posts, clinics, health centers, and hospital facilities. Lake Chilwa is used by people living on and around it as a source of drinking water, for bathing, and as a toilet. As a consequence, fecal contamination levels were high [17]. Of the total Malawian population, 59% and 10% had no sustainable access to improved sanitation and safe drinking water sources, respectively [18].

### Data collection aspects

Data collection was conducted from February to March 2016, using structured questionnaires. The assessment of the treatment cost of cholera to households and health facilities gathered data retrospectively, while that of the reactive immunization campaign used retrospective and prospective approaches. The treatment costs of cholera to households were elicited by interviewing the head of household or the main patient’s caregiver, while costs for health facilities were obtained by interviewing the health personnel, and reviewing cholera care registries. The reactive immunization campaign costs were extracted from programmatic documents, microplanning, budget, and financial reports of institutions that supported the implementation of the campaign. All cost data were presented in US dollars based on annual conversion rates from OANDA (http://www.oanda.com/currency/converter/) [19].

### Measurement of cholera costs to the household and health facility

Costs borne by households for the treatment of cholera were analyzed from a sample of 100 patients’ households. To minimize recall biases, eligible patients were cases presenting to cholera treatment centres with an infectious disease characterized by intense vomiting and profuse watery diarrhea, regardless of vaccination status, that received care over the previous five weeks. Collected data encompassed direct and indirect costs borne by patients’ households. Direct treatment costs included out-of-pocket medical and non-medical costs while indirect costs included wage losses due to the number of days absent from work for currently employed persons.

The treatment cost of cholera to the health facility was measured at selected four cholera treatment centers and one district hospital that managed cholera. Only direct costs, including staff, medicines, and consumables, were analyzed. The ingredient approach was used to estimate quantities and prices of items used to treat cholera. Interviews were also conducted with health personnel to investigate the time spent treating cholera patients. Details of the cost-of-illness study to household and health facility are available elsewhere [15].

### Measurement of immunization costs

The immunization campaign, implemented from February 16–22 and March 8–15, 2016, targeted 90,000 individuals aged more than one-year-old, including pregnant women. Costs incurred for the conduct of immunization activities were collected to assess both vaccine and delivery costs per fully immunized person. Data collected included vaccine, personnel, perdiems, material, equipment, transportation, rental, catering, operating costs, wasted vaccines, and miscellaneous costs. A detailed description of the costing assessment has been published elsewhere [14].

### Data management and analysis

#### Treatment costs of cholera to households and health facilities

The average treatment cost of cholera to households was estimated by computing the average cost among patients. The treatment cost of cholera per household was estimated as the sum of direct out-of-pocket expenses in medicines and consumables before hospitalization, plus direct and indirect costs borne during hospitalization. Direct medical and non-medical costs were estimated as the sum total of incurred costs before and during hospitalization, while indirect costs were only estimated for those who were working, based on self-reported total reduction of income due to the inactive period induced by the cholera episode.

The average treatment cost of cholera to health facilities was obtained by calculating the mean costs across three categories of patients treated for cholera (hospitalized for less than 12 h and discharged; hospitalized for more than 12 h and discharged; and deceased during hospitalization). The treatment cost by category of patients was obtained by adding up personnel, drugs, and consumables used to treat cases. The estimated cost for each category of personnel was based on time spent treating a cholera case and staff category income level. The total personnel cost was obtained by adding-up these estimates. Similarly, the cost of each drug and consumable used in treating a patient was obtained by multiplying the units of each input by its price. The total cost of drugs and consumables was then calculated as the total sum spent on these items by category of patients.

### Immunization costs and unit delivery costs per fully vaccinated person

The total incurred economic cost for the immunization campaign was obtained by adding-up financial and opportunity costs of capital resources. The financial cost was estimated as the sum total of costs incurred for all immunization related activities. The total cost for each immunization activity was obtained by adding up the total costs of all inputs used for that given activity. Total cost of a given input was calculated by multiplying quantities used by the corresponding unit price for recurrent inputs, and annualized before being accounted for some equipment such vaccine carriers and cold boxes. Opportunity costs covered some equipment and civil servants’ time. Civil servants’ time costs were accounted for by multiplying the corresponding daily wage of each of these human resources by the corresponding time involved. The unit economic cost of vaccine delivery per fully vaccinated person was estimated by dividing the total economic cost of the immunization campaign, excluding vaccine procurement and shipment, by the total number of people receiving the complete vaccine doses.

### Cost-effectiveness evaluation

The cost-effectiveness analysis was evaluated using VICE, the Vaccine Introduction Cost-Effectiveness calculator [20]. VICE is an Excel-based model for estimating the cost-effectiveness of vaccination programs. Though initially designed for the calculation of the cost-effectiveness of oral cholera vaccine introduction, the model is applicable to any disease interventions in various settings. Upon entry of needed individual parameters into the model, three metrics, including the net cost per: 1) case; 2) death; and 3) DALY averted were automatically generated. The net cost was estimated as the total reactive immunization costs minus household and health facility cost-of-illness averted by immunization. The denominators for the cost-effectiveness ratios were estimated for the entire immunization program. The estimation of DALYs took into account both morbidity (years of healthy life lost to disability, YLD) and mortality (years of life lost to premature mortality, YLL) due to cholera. Consistent with previous research, the following five-step equations were used to calculate the total DALYs averted [21, 22].

1. YLD averted_i,t_ = {[(1-CFR_i_)*VEff_t_*Cover_i_*Inc_i_]*Length*DALY9 weight}
2. YLL averted_i,t_ = {[(CFR_i_*VEff_t_*Cover_i_*Inc_i_)/0.03]*[1-exp(-0.03*LExp)]}
3. DALYs averted_i,t_ = YLD averted_i,t_ + YLL averted_i,t_
4. Total DALYs averted_i,t_ = ${\sum }_{\mathrm{t}=0}^{\mathrm{Dur}}\left({\mathrm{DALYs }}_{\mathrm{i},\mathrm{t}}\right)$/(1 + 0.03)^t^
5. Cost-effectiveness ratio = Vaccination cost/total DALYs averted

With *VEff* = vaccine efficacy against cholera disease; *Cover* = vaccine coverage for two doses; *CFR* = case fatality ratio*; Inc.* = cholera disease incidence*; DALY weight* = disability weight associated with morbidity from cholera; *Length* = average duration of cholera in days*; LExp* = life expectancy at infection onset*; Dur* = duration of the vaccine’s protective effect for the observed period; *t* is the time in years, and *i* indicates the subpopulation i. However, the analysis conducted in the current study only looked at the global population since detailed data of various age subpopulations have not been collected. Both costs and effectiveness indicators were discounted at a real interest rate of 3%.

The vaccination coverage survey conducted two weeks apart from the campaign reported that 68 570 individuals were vaccinated during the 1st round, and 54 808 received two doses of Shanchol vaccine, with a 20% drop-out rate [23]. In view of this, a static cohort consisting of 54,808 individuals fully vaccinated out of 90,000 people targeted by the campaign was considered in the analysis. Cost-effectiveness metrics were first computed from the base-case scenario by running a model that incorporated economic, demographic, and epidemiological data. The metrics were then re-estimated, incorporating indirect protection effects, and assessed against gross national product (GDP) thresholds. The immunization campaign was considered “very cost-effective” and “cost-effective” if the net cost per DALY averted was less than one time and three times GDP per capita, respectively. We further investigated uncertainties surrounding cost-effectiveness ratios by conducting one-way sensitivity analyses.

### Indirect effects of vaccine

Numerous studies have shown that cholera vaccine can provide indirect protection to unvaccinated individuals through reducing disease transmission [21, 24–26], an effect dependent on immunization coverage. For example, in Bangladesh the overall vaccine protection was 93% at an immunization coverage level of 50% [24]. A similar finding was reported by Ali et al. 2013 in India [26]. Research also showed a reverse correlation between cholera incidence in vaccine recipients and vaccine coverage. Based on these findings, epidemiological models predicting vaccine effectiveness as a function of vaccine coverage have been developed [24]. The epidemiological sub-study which was conducted alongside the cost-effectiveness evaluation of OCV campaign in Malawi did not assess the indirect protection conferred to unvaccinated. Because of this, we estimated model outcomes with indirect protection by applying the overall protective efficacy level reported by the above mentioned published papers [24, 26], assuming a total protective efficacy of 93% (95% CI 82%–99%) for the corresponding double dose vaccine coverage rate [24].

### Cost-effectiveness model input parameters

Table 1 presents base-case values and uncertainty ranges of all input parameters used to model cost-effectiveness ratios. Parameters were mostly derived from the in-country data collection and analysis. When a primary source of data was not available, review articles were the preferred sources for input parameters and uncertainty ranges. When data from review papers were unavailable for a specific parameter, data from individual articles were considered.

**Table 1:** Model input parameters with uncertainty ranges in brackets

	Central	Uncertainty	Source of data	Assumptions
Parameters	value	range	Central value	Uncertainty ranges (min – max)
Economic data				
Vaccine purchase price per fully immunized, 2016 US$	3.7	–	Derived from the analysis	–
Vaccine delivery cost per fully immunized, 2016 US$	3.6	[1.1–3.6]	Ilboudo et al. 2017 [14]	(Mogasale et al. 2016 [27]—Ilboudo et al. 2017 [14]
Cost of cholera to patients and households, 2016 US$	65.6	[43.0–134.0]	Ilboudo et al. 2017 [15]	(Schaetti et al. 2012 [29]—Poulos et al. 2012 [28]
Cost of cholera to health facilities, 2016 US$	59.7	[30.0–61.0]	Ilboudo et al. 2017 [15]	(Poulos et al. 2012 [28]—Schaetti et al. 2012 [29]
GDP per capita, 2016 US$	372.0	–	World Bank 2017 [31]	–
Discount rate (%)	3.0	[1.0–5.0]	WHO 2008 [30]	(Min and max from WHO, 2008 [30])
Epidemiological data				
Cholera incidence (Inc, cases per 1,000)	4.0	[3.0–5.0]	Sauvageot et al. 2017 [32]	(Min and max from Sauvageot et al. 2017 [32])
Case fatality rate (*CFR*, %)	2.6	[1.4–6.7]	M’Bangombé, 2017 [33]	(Min and max from M’Bangombé, 2017 [33])
Vaccine protective duration (Dur, years)	5.0	[3.0–5.0]	Bhattacharya et al. 2013 [9]	(Min and max from Bhattacharya et al. 2013 [9])
Length of illness (Length, days)	5.0	[4.0–7.0]	Ilboudo et al. 2017 [15]	(Poulos et al. 2012 [28]—Schaetti et al. 2012 [29])
DALY weight (DALY weight)	0.2	[0.1–0.3]	Salomon et al. 2012 [36]	(Min and max from Salomon et al. 2012 [36])
Vaccine Efficacy (VEff, no indirect protection)	58.0	[42.0–69.0]	Bi et al. 2017 [35]	(Min and max from Bi et al. 2017 [35])
Vaccine Efficacy (VEff, with indirect protection)	93.0	[82.0–99.0]	Longini et al. 2007 [24]	(Min and max from Longini et al. 2007 [24])
Campaign coverage rate (%, without indirect protection)	58.0	[53.0–91.0]	MSF 2016 [4]	(Min and max from MSF, 2016 [34])
Demographic				
Population	90 000.0	–	–	–
Life expectancy at infection (LExp, years)	58.0	[50.0–60.0]	WHO, 2016 [37]	(Min and Max from WHO, 2016 [37])

### Economic parameters

Base-case values for the costs of cholera to patients’ households, health facilities, and vaccine delivery originated from costing studies we conducted in Malawi. The mean treatment costs of cholera to patients’ households and health facilities amounted to US$65.6 and US$59.7, respectively [15]. Vaccine delivery cost per fully vaccinated person was estimated at US$3.6 [14]. For these three input parameters, except for the upper value for uncertainty range of vaccine delivery cost per fully vaccinated person taken from Mogasale et al. 2016 [27], central values from published studies with similar methods were used as uncertainty ranges. As our estimate of vaccine delivery cost per fully vaccinated person was higher than other publications, we used it as the upper value for the uncertainty range. In addition, the base-case value for cholera illness duration was taken from the cost-of-illness study [15], and uncertainty ranges were taken from comparable studies [28, 29]. Vaccine price has been set internationally at US$3.7 for two doses. Due to this, sensitivity analyses were not performed on the two-dose vaccine price. The uncertainty range for the discount rate was set at 1–5% to conform with the WHO guide for standardization of economic evaluations [30]. Finally, data on GDP originated from the World Bank’s open data source [31].

### Vaccine effects

The epidemiological study, also conducted alongside the immunization campaign, provided base-case values for cholera incidence [32], case fatality ratio [33], and vaccine coverage [34]. The vaccine protective efficacy, without indirect protection, was derived from a review article indicating an average 58% protective efficacy of the vaccine at five years, with uncertainty ranges from the same study [35]. With the demonstrated five-year vaccine protective efficacy duration for two doses, the vaccine protection duration was set at five years, with uncertainty from three to five years consistent with literature [9, 11]. The weighted vaccine protective efficacy from Machinga, Phalombe and Zomba were computed and used in the base-case scenario analysis, while the overall vaccine protective efficacy, coverage level, and uncertainty ranges, including indirect protection, were taken from Longini et al. 2007 [24]. In the absence of disability weights specific to cholera, those for diarrheal diseases were used to approximate YLL to disability because of cholera in Malawi. The disability weight for moderate diarrhea was used as a base-case value, while those for mild and severe diarrhea were used for sensitivity analyses [36].

### Demographic data

Regarding the demographic data, the World Health Statistics 2016 data maintained by the WHO provided the base-case value for life expectancy in Malawi and uncertainty ranges [37].

## Results

### Cost-effectiveness outcomes

Assuming vaccine efficacy of 58% i.e. base-case scenario, the reactive immunization campaign would have resulted in 636 cases (619 non-fatal and 17 fatal cases) and 430 DALYs averted. A total of 172 and 6 631 doses of Shanchol vaccine would be required to avert a case and death from cholera, respectively. Under this scenario, the estimated amount needed to implement the 2-dose reactive immunization campaign with Shanchol would total US$397 358. The campaign would have contributed to averting cholera-related treatment costs of US$79 789 to households and health facilities, and the net immunization program costs would have been worth US$317 569 (Table 2).

**Table 2:** Key immunization program cost-effectiveness outcomes

Parameters	Base case	With indirect protection
Effects		
Total number of non-fatal cases averted	619	993
Total number of deaths averted	17	27
Total number of cases averted	636	1 020
Total DALYs averted	430	690
Costs		
Immunization program costs	397 358	397 358
Total costs averted (households plus health facilities)	79 789	127 938
Net program costs	317 569	269 420
Cost-effectiveness ratios		
Net cost per death averted	19 212	10 165
Net cost per case averted	500	264
Net cost per DALY averted	738	391
Vaccine requirements		
Doses per death averted	6 631	4 136
Doses per case averted	172	108
GDP thresholds		
Very cost-effective (GDP/capita)	372	372
Cost-effective (3*GDP/capita)	1116	1116

Assuming vaccine efficacy of 93% i.e. with consideration of indirect protection, 1 020 cases (993 non-fatal and 27 fatal cases) and 690 DALYs could have been averted. In total 108 and 4 136 doses of Shanchol vaccine would be required to avert a case, and a death from cholera, respectively. Under this second scenario, the campaign would have contributed to averting cholera-related treatment costs of US$127 938 to households and health facilities, and the net immunization program costs would have been US$269 420.

The net cost per DALY averted under the base-case scenario, estimated at US$738 suggested that the reactive mass immunization campaign was cost-effective. The campaign remained just cost-effective when indirect protection was incorporated, at a net cost per DALY averted of US$391. However, the incorporation of indirect protection was translated into improved health and cost outcomes, with a 60% increase in total costs averted and a reduction of the net program costs of approximately 15%.

### Sensitivity analysis

**Fig. 1:**
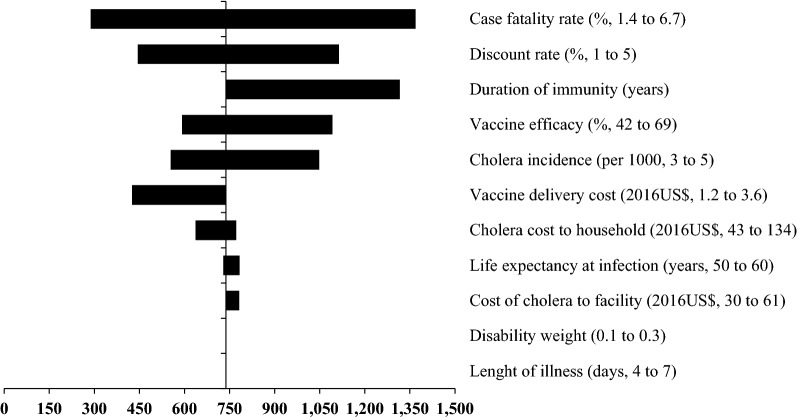
Tornado diagram of univariate sensitivity analysis of net cost per DALY averted without indirect protection

Three input parameters, including case fatality rate, discount rate and duration of immunity had an influence on the net cost per DALY averted under the base-case scenario i.e. without indirect protection (Fig. 1). Parameters such as vaccine delivery costs, cost-of-illness (household and health facility), life expectancy at infection, length of illness and disability weight showed relatively less important influences on the net cost per DALY averted.

**Fig. 2:**
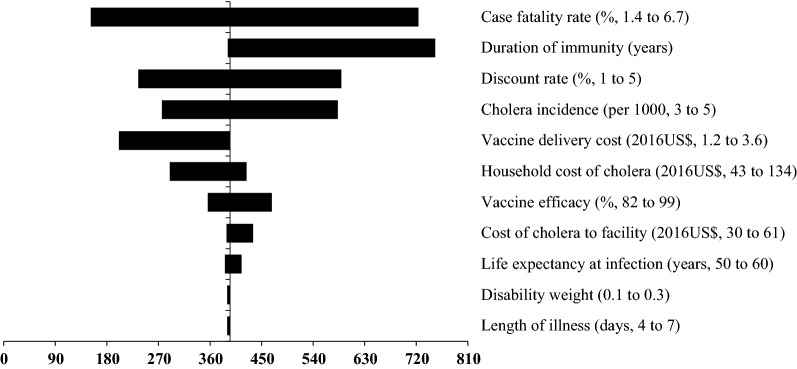
Tornado diagram of univariate sensitivity analysis of net cost per DALY averted with indirect protection

The incorporation of indirect protection further showed that the above mentioned three inputs parameters had a much larger influence on cost-effectiveness estimates, showed by larger intervals (Fig. 2).

 Regardless of incorporation of indirect protection, the net costs per case and death averted were also most sensitive to six most influential input parameters, including cholera incidence, vaccine efficacy, vaccine delivery cost per fully immunized person, cost-of-illness of cholera to households, duration of immunity, and case fatality rate (Additional files 1: Tables S1 and S2).

## Discussion

This study investigated the cost-effectiveness of a reactive immunization campaign using Shanchol in Malawi. Despite the growing literature on the cost-effectiveness of Shanchol use, evidence in reactive situations is scarce. The contribution of this paper to the literature lies in the fact that it provides further useful decision-making evidence on the value for money of Shanchol use for immunization campaigns. To the best of our knowledge, most cost-effectiveness evaluations with Shanchol vaccine were conducted in the Asian continent [22, 38, 39] while our study was conducted in one of the African country which has long been affected by cholera. Therefore, we think that the demonstration made in this study may be a catalyst together with other studies to accelerate the accumulation of evidence for guiding policy-making towards a wider use of OCVs.

The findings of this study show that the reactive immunization campaign was cost-effective even without incorporation of indirect protection. The intervention did not become very cost-effective even when indirect protection was considered. This finding is consistent with the results from Jeuland et al. 2009 who found that OCV campaigns in North Jakarta, Indonesia and Matlab, Bangladesh were just cost-effective when indirect protection was incorporated [21]. However, indirect protection importantly improved immunization outcomes by increasing the number of cases and deaths averted. This finding is consistent with a previous cost-effectiveness analysis demonstrating the potentiating effects of indirect protection on immunization outcomes in Bangladesh, India, Indonesia, and Mozambique [21]. Our study finding is also consistent with that from Kim et al. 2011 in Zimbabwe though this study used a lower vaccine cost estimate than that of our study [40].

The results also showed that the net cost per DALY averted was sensitive to cholera-associated incidence and mortality rate. This finding is consistent with a previous study that reported the sensitivity of DALYs averted to changes in cholera-associated severity and fatality levels [22]. These findings suggest that cholera immunization campaigns may be appropriate in settings with high cholera incidence and mortality. We likely underestimated disease burden by relying on hospital data, since cholera cases and deaths may also occur in the community.

Consistent with the literature [21], the net cost per DALY averted also was influenced by changes in vaccine delivery costs per fully vaccinated person. Moreover, household and health facility costs averted by immunization may have also been much higher than what was presented in this study if a larger range of costs had been accounted for, including costs for setting-up cholera treatment centers and costs of mortality from cholera [15].

## Limitations

Our study has limitations. First, the cost-effectiveness modeling was built upon a static cohort. Although this modeling may provide useful information, it does not consider the complex transmission dynamics of cholera. Research showed that not all vaccinated people will be immune to cholera during the analytical horizon [41]. This may have led to an overestimation of potential benefits of the intervention, particularly because cholera vaccine protection wanes over time [29]. Second, the cost-effectiveness ratios we presented may have been distorted by uncertainties relative to data gaps. For example, we relied on vaccine indirect protection estimates from Bangladesh, and indirect protection may vary by setting [21]. Third, we modeled outcomes for the entire population rather than by age cohorts since age specific data were absent for many parameters. Age-specific analyses may identify groups for which vaccine would be a more efficient intervention, such as children [21]. Fourth, cost-effectiveness calculations were based on a two-dose regimen as recommended by WHO [42]. VICE cost-effectiveness model has been parametrized to take into account either a single- or a two-dose vaccine administration. Because of this, it has not been possible to take into account both single- and two-dose regimen effects in the same modelling using VICE for the calculations of cost-effectiveness ratios. Since reasonable evidence suggests that a single dose of oral cholera vaccine provides moderate protection from cholera [43–46], the current findings could have understated the effects and cost-effectiveness ratios. Finally, some costs were not assessed. Missing costs may have led the campaign to appear more cost-effective than was reported.

## Conclusion

The analysis showed in this study suggests that the immunization campaign can be considered cost-effective compared to the absence of immunization. However, due to imprecisions related to model input parameters, caution must be used when extrapolating the results to other settings.

## Supplementary Information

**Additional file1: Table S1:** Effect of individual parameters on net cost per case averted.** Table S2:** Effect of individual parameters on net cost per death averted.

## Data Availability

The datasets used and/or analyzed during the current study are available from the corresponding author on reasonable request.

## References

[1] Deen JL, von Seidlein L, Sur D, Agtini M, Lucas ME, Lopez AL (2008). The high burden of cholera in children: comparison of incidence from endemic areas in Asia and Africa. PLoS Negl Trop Dis.

[2] Kirigia JM, Sambo LG, Yokouide A, Soumbey-Alley E, Muthuri LK, Kirigia DG (2009). Economic burden of cholera in the WHO African region. BMC international health and human rights.

[3] WHO. Cholera vaccines: WHO position paper. 2010 Contract No.: 85.

[4] Clemens J, Holmgren J (2014). When, how, and where can oral cholera vaccines be used to interrupt cholera outbreaks?. Curr Top Microbiol Immunol.

[5] WHO. Prevention and control of cholera outbreaks: WHO policy and recommendations. Global Task Force on Cholera Control. 2011.

[6] WHO/UNICEF. Progress on Drinking Water and Sanitation: 2012 Update. 2012.

[7] WHO. WHO Prequalified Vaccines Geneva, Switzerland: WHO; 2016 [12/13/2016]. https://extranet.who.int/gavi/PQ_Web/.

[8] Sur D, Kanungo S, Sah B, Manna B, Ali M, Paisley AM (2011). Efficacy of a low-cost, inactivated whole-cell oral cholera vaccine: results from 3 years of follow-up of a randomized, controlled trial. PLoS Negl Trop Dis.

[9] Bhattacharya SK, Sur D, Ali M, Kanungo S, You YA, Manna B (2013). 5 year efficacy of a bivalent killed whole-cell oral cholera vaccine in Kolkata, India: a cluster-randomised, double-blind, placebo-controlled trial. Lancet Infect Dis.

[10] Martin S, Lopez AL, Bellos A, Deen J, Ali M, Alberti K (2014). Post-licensure deployment of oral cholera vaccines: a systematic review. Bull World Health Organ.

[11] Kabir S (2014). Critical analysis of compositions and protective efficacies of oral killed cholera vaccines. Clinical Vaccine Immunol.

[12] Ali M, You YA, Kanungo S, Manna B, Deen JL, Lopez AL (2015). Assessing different measures of population-level vaccine protection using a case-control study. Vaccine.

[13] Desai SN, Pezzoli L, Alberti KP, Martin S, Costa A, Perea W (2017). Achievements and challenges for the use of killed oral cholera vaccines in the global stockpile era. Human Vaccines Immunotherapeutics.

[14] Ilboudo PG, Le Gargasson JB (2017). Delivery cost analysis of a reactive mass cholera vaccination campaign: a case study of Shanchol vaccine use in Lake Chilwa, Malawi. BMC Infect Dis.

[15] Ilboudo PG, Huang XX, Ngwira B, Mwanyungwe A, Mogasale V, Mengel MA (2017). Cost-of-illness of cholera to households and health facilities in rural Malawi. PLoS ONE.

[16] Malawi National Statistical Office. GeoHive - Malawi population statistics Malawi: Geohive; 2016 [11/30/2016]. http://www.geohive.com/cntry/malawi.aspx.

[17] Khonje A, Metcalf CA, Diggle E, Mlozowa D, Jere C, Akesson A (2012). Cholera outbreak in districts around Lake Chilwa, Malawi: lessons learned. Malawi Medical J.

[18] WHO. World health statistics (2016). monitoring health for the SDGs, sustainable development goals.

[19] OANDA. 1996–2016 OANDA 2016 [17/11/2016]. https://www.oanda.com/lang/fr/.

[20] Troeger C, Chao D, Sack D (2014). Vaccine Introduction Cost-Effectiveness Calculator (VICE).

[21] Jeuland M, Cook J, Poulos C, Clemens J, Whittington D, Group DCES (2009). Cost-effectiveness of new-generation oral cholera vaccines: a multisite analysis. Value In Health J Int Society Pharmacoeconomics Outcomes Res.

[22] Troeger C, Sack DA, Chao DL (2014). Evaluation of targeted mass cholera vaccination strategies in Bangladesh: a demonstration of a new cost-effectiveness calculator. Am J Trop Med Hyg.

[23] Médecins Sans Frontières. Cholera outbreak response Lake Chilwa south-east Region districts of Machinga, Phalombe and Zomba. 2016.

[24] Longini IM, Nizam A, Ali M, Yunus M, Shenvi N, Clemens JD (2007). Controlling endemic cholera with oral vaccines. PLoS Med.

[25] Sack DA (2006). Herd protection and herd amplification in cholera. J Health Popul Nutr.

[26] Ali M, Sur D, You YA, Kanungo S, Sah B, Manna B (2013). Herd protection by a bivalent killed whole-cell oral cholera vaccine in the slums of Kolkata, India. Clinical infectious diseases Official Publication Infectious Diseases Society America.

[27] Mogasale V, Ramani E, Wee H, Kim JH (2016). Oral Cholera vaccination delivery cost in low- and middle-income countries: an analysis based on systematic review. PLoS Negl Trop Dis.

[28] Poulos C, Riewpaiboon A, Stewart JF, Clemens J, Guh S, Agtini M (2012). Costs of illness due to endemic cholera. Epidemiol Infect.

[29] Schaetti C, Weiss MG, Ali SM, Chaignat CL, Khatib AM, Reyburn R (2012). Costs of illness due to cholera, costs of immunization and cost-effectiveness of an oral cholera mass vaccination campaign in Zanzibar. PLoS Negl Trop Dis.

[30] World Health Organization, editor. WHO guide for standardization of economic evaluations of immunization programmes. Geneva: WHO; 2008.

[31] World Bank. Workd http://data.worldbank.org/.

[32] Sauvageot D, Munier A, Saussier C. Impact Study of Oral Cholera Vaccine Campaign, Machinga and Zomba Districts, Malawi: re-analysis of cholera incidence data. 2017.

[33] M'Bangombe M (2017). Cholera in Malawi.

[34] MSF. Oral cholera vaccintion campaign on Lake Chilwa and its three districts (Machinga, Phalombe, Zomba), Malawi. Paris, France: MSF, 2016.

[35] Bi Q, Ferreras E, Pezzoli L, Legros D, Ivers LC, Date K (2017). Protection against cholera from killed whole-cell oral cholera vaccines: a systematic review and meta-analysis. Lancet Infect Dis.

[36] Salomon JA, Vos T, Hogan DR, Gagnon M, Naghavi M, Mokdad A (2012). Common values in assessing health outcomes from disease and injury: disability weights measurement study for the Global Burden of Disease Study 2010. Lancet.

[37] WHO. Life expectancy at birth (years), 2000–2015. Both sexes: 2015 Geneva, Switzerland2016 [cited 2017 22/02]. http://gamapserver.who.int/gho/interactive_charts/mbd/life_expectancy/atlas.html.

[38] Smalley HK, Keskinocak P, Swann J, Hinman A (2015). Optimized oral cholera vaccine distribution strategies to minimize disease incidence: A mixed integer programming model and analysis of a Bangladesh scenario. Vaccine.

[39] Levin A, Maskery B, DeRoeck D, al. e. An investment case for the accelerated introduction of oral cholera vaccines. . International Vaccine Institute, 2012.

[40] Kim SY, Choi Y, Mason PR, Rusakaniko S, Goldie SJ. Potential impact of reactive vaccination in controlling cholera outbreaks: an exploratory analysis using a Zimbabwean experience. South African medical journal = Suid-Afrikaanse tydskrif vir geneeskunde. 2011;101(9):659–64. 21920160

[41] Fung IC (2014). Cholera transmission dynamic models for public health practitioners. Emerging Themes Epidemiol.

[42] Desai SN, Pezzoli L, Martin S, Costa A, Rodriguez C, Legros D (2016). A second affordable oral cholera vaccine: implications for the global vaccine stockpile. Lancet Global health.

[43] Tembo T, Simuyandi M, Chiyenu K, Sharma A, Chilyabanyama ON, Mbwili-Muleya C (2019). Evaluating the costs of cholera illness and cost-effectiveness of a single dose oral vaccination campaign in Lusaka, Zambia. PLoS ONE.

[44] Azman AS, Parker LA, Rumunu J, Tadesse F, Grandesso F, Deng LL (2016). Effectiveness of one dose of oral cholera vaccine in response to an outbreak: a case-cohort study. Lancet Global health.

[45] Ferreras E, Chizema-Kawesha E, Blake A, Chewe O, Mwaba J, Zulu G (2018). Single-Dose Cholera Vaccine in Response to an Outbreak in Zambia. N Engl J Med.

[46] Qadri F, Wierzba TF, Ali M, Chowdhury F, Khan AI, Saha A (2016). Efficacy of a Single-Dose, Inactivated Oral Cholera Vaccine in Bangladesh. N Engl J Med.

