# Supplementary material for: Cost-effectiveness of a reactive oral cholera immunization campaign using Shanchol™ in Malawi
Source: Cost Eff Resour Alloc. 2021 Mar 10;19:17. doi: 10.1186/s12962-021-00270-y (PMC7945304; doi:10.1186/s12962-021-00270-y)
Supplement: Supplementary file 1 — Additional file1: Table S1: Effect of individual parameters on net cost per case averted. Table S2: Effect of individual parameters on net cost per death averted. [file 12962_2021_270_MOESM1_ESM.docx]

**Table S1: Effect of individual parameters on net cost per case averted**

|  | **Base case** | | **With indirect protection** | |
| --- | --- | --- | --- | --- |
| **Parameter** | **Minimum** | **Maximum** | **Minimum** | **Maximum** |
| Lenght of illness (*Lenght*, days) | 500 | 500 | 264 | 264 |
| Disability weight (*DALY weigh*t) | 500 | 500 | 264 | 264 |
| Life expectancy (LExp, years) | 500 | 500 | 264 | 264 |
| Discount rate (%) | 500 | 500 | 264 | 264 |
| Case fatality rate (*CFR*, %) | 500 | 500 | 264 | 264 |
| Duration of immunity (*Dur*, years) | 499 | 530 | 264 | 524 |
| Health facility cost of cholera (2016 USD) | 432 | 523 | 263 | 294 |
| Cholera incidence (*Inc*, cases per 1000) | 288 | 500 | 186 | 394 |
| Vaccine efficacy (*Veff*, %) | 375 | 709 | 241 | 317 |
| Household cost of cholera (2016 USD) | 401 | 739 | 196 | 287 |
| Vaccine delivery cost (2016 USD) | 500 | 918 | 202 | 461 |

**Table S2: Effect of individual parameters on net cost per death averted**

|  | **Base case** | | **With indirect protection** | |
| --- | --- | --- | --- | --- |
| **Parameter** | **Minimum** | **Maximum** | **Minimum** | **Maximum** |
| Lenght of illness (Lenght, days) | 19 245 | 19 245 | 10 165 | 10 165 |
| Disability weight (DALY weight) | 19 245 | 19 245 | 10 165 | 10 165 |
| Life expectancy (LExp, years) | 19 245 | 19 245 | 10 165 | 10 165 |
| Discount rate (%) | 19 245 | 19 245 | 10 165 | 10 165 |
| Health facility cost of cholera (2016 USD) | 19 202 | 20 395 | 10 122 | 11 315 |
| Household cost of cholera (2016 USD) | 16 622 | 20 122 | 7 542 | 11 042 |
| Vaccine delivery cost (2016 USD) | 11 088 | 19 245 | 7 787 | 17 712 |
| Cholera incidence (Inc, cases per 1000) | 14 430 | 27 269 | 7 166 | 15 162 |
| Vaccine efficacy (Veff, %) | 15 407 | 28 415 | 9 256 | 12 176 |
| Duration of immunity (Dur, years) | 19 245 | 35 292 | 10 165 | 20 159 |
| Case fatality rate (CFR, %) | 7 468 | 35 740 | 3 945 | 18 877 |
